# The DNA Polymerase ʱ-Primase Complex: Multiple Functions and Interactions

**DOI:** 10.1100/tsw.2003.05

**Published:** 2003-03-17

**Authors:** Marco Muzi-Falconi, Michele Giannattasio, Marco Foiani, Paolo Plevani

**Affiliations:** Dipartimento di Genetica e di Biologia dei Microrganismi, Universit? degli Studi di Milano, Via Celoria 26, 20133 Milano, Italy

**Keywords:** DNA polymerase a, DNA primase, DNA replication, checkpoints, DNA repair, chromatin, yeast

## Abstract

DNA polymerase _ (pol _) holds a special position among the growing family of eukaryotic DNA polymerases. In fact, pol _ is associated with DNA primase to form a four subunit complex and, as a consequence, is the only enzyme able to start DNA synthesis de novo. Because of this peculiarity the major role of the DNA polymerase _-primase complex (pol-prim) is in the initiation of DNA replication at chromosomal origins and in the discontinuous synthesis of Okazaki fragments on the lagging strand of the replication fork. However, pol-prim seems to play additional roles in other complex cellular processes, such as the response to DNA damage, telomere maintenance, and the epigenetic control of higher order chromatin assembly.

## INTRODUCTION

The maintenance of genome stability is of crucial importance for all living organisms. DNA must be replicated with high fidelity every cell cycle and different types of DNA damage must efficiently be repaired through the correct interplay among several DNA transactions (replication, repair, recombination, and transcription). Nonetheless, during DNA replication it may be more convenient for the cell to synthesize DNA across a lesion at the expense of fidelity rather than abort the replication process itself. Indeed, many novel DNA polymerases (pols) discovered in the last few years are involved in translesion DNA synthesis[1,2].

Pol α was the first polymerase identified in eukaryotic cells and for several years it was thought to be the only pol required for chromosomal DNA replication, while pol α and pol α were, respectively, the repair enzyme and the pol required for mitochondrial DNA replication. Later pol α and pol α were identified as additional pols required for nuclear DNA replication and a DNA primase activity was found to be associated with pol α, providing an explanation on how DNA replication can be initiated[1,3]. In fact, all known pols lack the capacity to start DNA chains de novo on single-stranded (ss) templates and require the 3'-end of a pre-existing primer molecule. Several strategies evolved during evolution to solve this problem, but the more frequently used by viruses, prokaryotes, and eukaryotes is by far the use of a short RNA molecule synthesized by DNA primase that, in this view, can be considered as a specialized RNA polymerase required for initiation of DNA chains[3,4,5].

## STRUCTURE OF THE EUKARYOTIC POL-PRIM COMPLEX

In all eukaryotic organisms analyzed to date, pol-prim is a four subunit complex (Fig. 1) evolutionary conserved from yeast to man and each subunit is essential for cell viability[3,4].

The largest, 180 kDa, polypeptide (p180) shows DNA polymerase activity *in situ* after SDS-PAGE electrophoresis and is, therefore, the catalytic pol α subunit[6]. The smallest subunit (p48) is sufficient to synthesize *in vitro* the short RNA primers utilized for elongation by pol α and, therefore, corresponds to the catalytic DNA primase subunit[7].The p58 polypeptide forms a tightly bound subcomplex with p48 and is generally considered as the second primase subunit, even though its physiological role is still poorly defined. It has been suggested that: (1) p58 helps to stabilize the thermolabile nature of the catalytic activity of p48 and, because of its intimate association with the NTP binding site, may facilitate optimal RNA primer synthesis[7]; (2) p58 is required for productive interaction between p48 and p180[8], possibly playing an essential role in the transition between RNA primers synthesis and their subsequent elongation by pol α; (3) p58 contains a nuclear localization signal that appears to be required for translocation of p48 within the nucleus through a piggy-back binding transport mechanism[9]. No enzymatic activity has been found to be associated with the fourth polypeptide of the complex, also called B subunit[3]. The human B subunit was proposed to act as molecular tether during DNA replication, because it mediates the *in vitro* interaction between human pol-prim and T antigen bound to the SV40 origin of replication[10]. Accordingly, genetic studies in *Saccharomyces cerevisiae* indicate that B subunit plays its essential role at a very early stage of the replication process, since it executes its function before the dNTP polymerization step, which is sensitive to inhibition by hydroxyurea[11]. The apparent molecular mass of B subunit is ranging from ~70 kDa in mammalian cells to ~90 kDa in budding yeast[2,11]. This variability can be ascribed to both the different size of the deduced gene products, and to differential post-translational modifications (see below).

Based on sequence homology and structural similarities, pols have been grouped in five families known as A, B, C, X, and Y and pol α belongs, together with the other replicative pols δ and ε, to family B[2]. Based on the pol α sequence, six highly conserved regions (I to VI) have been identified among eukaryotic, prokaryotic, and viral pols and, although pols from different families are structurally quite different, they fold into a conformation resembling a human right-hand composed of three distinct domains designed as palm, thumb, and fingers. Region IV is positioned at the N-terminus, while the highly conserved regions I and II are located in the palm domain and contains the catalytically important amino acid residues.

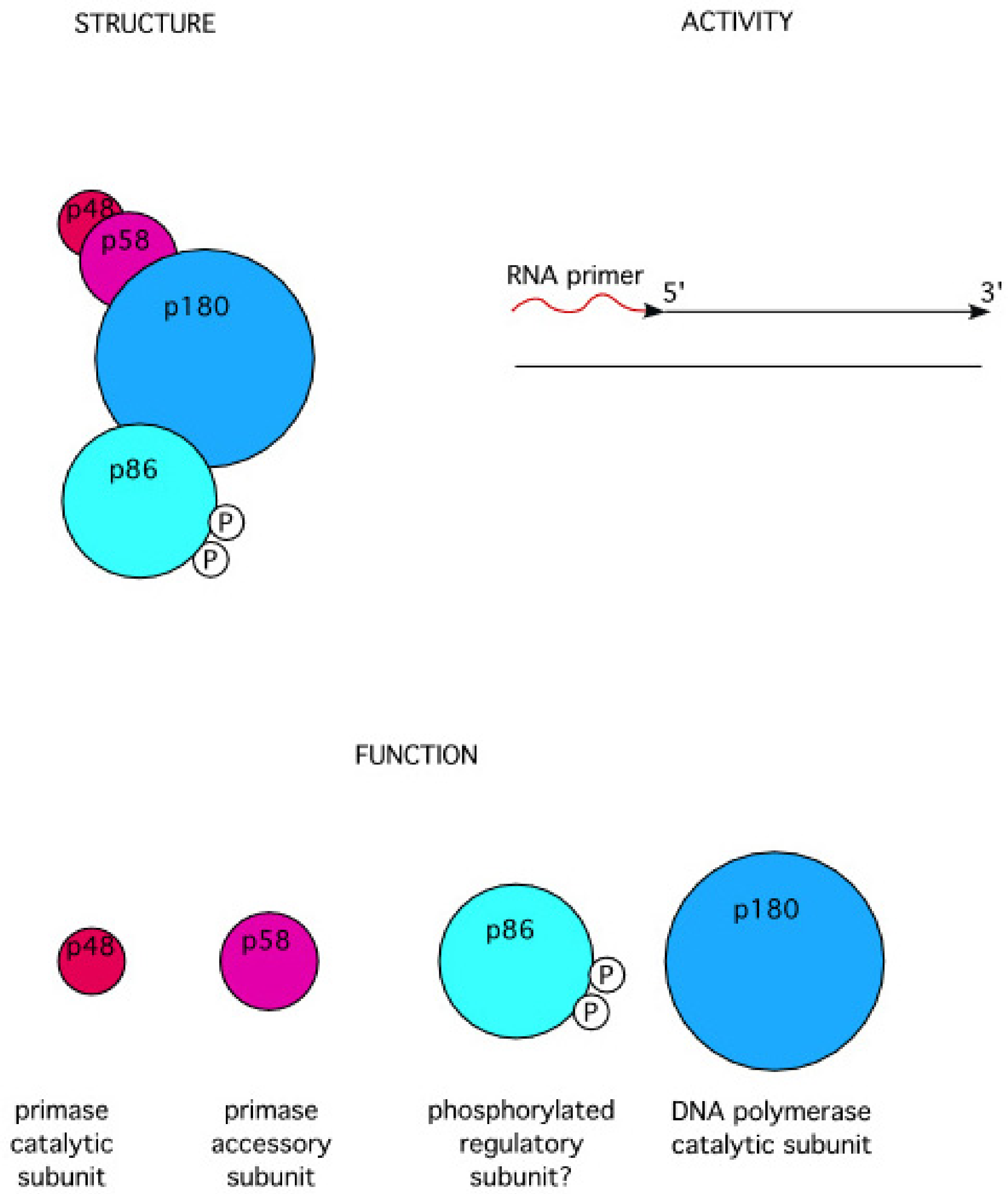

**FIGURE 1**. Structure and function of the pol-prim complex. The scheme represents the structure and function of the pol-prim complex in *S. cerevisiae* cells (see text for details).

Conserved region V is located within the thumb domain, and regions III and VI are located in the fingers. While the palm domain can be superimposed among members of different families, the thumb and fingers domains are unrelated among the family members, even though they function similarly using analogous structural elements.

Crystallographic information on eukaryotic DNA primase is still lacking and the only high resolution structure available is that of primase from the hyperthermophilic archaeon *Pyrococcus furiosus (Pfu)* which is closely related to the eukaryotic enzyme[12]. *Pfu*-primase is folded into two globular and tightly packed domains. The first domain contains most of the residues conserved within eukaryotes and archea, including the three invariant negatively charged aspartate residues essential for primase activity[13] which would be positioned similarly to the "catalytic triad" of DNA pols[14]. The second domain is highly variable in length and sequence even within archea, and it might mediate interaction with proteins functionally equivalent to the eukaryotic p58 primase subunit. *Pfu* and eukaryotic primases are likely to be members of the X family of pols[2,13,15), which includes enzymes as diverse as pol β and terminal transferase, while they do not appear to share structural similarity with *Escherichia coli* primase[16]. This last finding is quite surprising since *E. coli* and eukaryotic primases share a number of mechanistic and biochemical properties.

## REACTION MECHANISMS OF THE POL-PRIM COMPLEX AT THE REPLICATION FORK

Since pol-prim is required for initiation of DNA synthesis, the complex has to be recruited at chromosomal origins of replication. Although pol-prim catalyzes the first biosynthetic reaction in the replication process, its action must follow the step-wise assembly of a multiprotein machinery known as the prereplicative complex (pre-RC)[17]. Most of the steps required for pre-RC formation have been sorted out and the succession of events at a *S. cerevisiae* origin is summarized in Fig. 2.

The Origin Recognition Complex (ORC) marks the chromosomal origins throughout the cell cycle acting as a landing platform to promote a cascade of protein-protein interactions. From late M to the G1 phase of the cell cycle, the Cdc6 and Cdt1 proteins act as the helicase recruiter loading the Mini-Chromosome Maintenance (MCM) complex which is the putative replicative DNA helicase[18]. Cdc45 and Sld3 then join the pre-RC and the subsequent activation of S-Cdks and the Cdc7-Dbf4 protein kinases facilitates origin unwinding and the association of the ss-DNA binding protein RP-A[19,20]. Finally, pol-prim and the other replicative pols are recruited to origins through a step likely requiring Dpb11 and Sld2[21]. After pol-prim becomes associated with the initiation complex at the origins the primase subunit starts to synthesize a short RNA primer 7–10 nucleotides (nt) long which is then elongated by a stretch of 20–30 deoxyribonucleotides by the pol α polypeptide. After the synthesis of this RNA-DNA hybrid molecule (also called initiator DNA) the pol-prim complex is substituted by pol δ[22,23], and perhaps pol ε[24], which continues the processive synthesis of the leading strand[3]. This switch of pols relocates the pol-prim complex on the retrograde arm of the replication bubble where repeatedly synthesizes the RNA-DNA initiator molecules required to prime each Okazaki fragment during discontinuous synthesis of the lagging strand. The maturation of the Okazaki fragments to their final length of approximately 200 nt also requires the switch of the pol-prim complex with pol δ or pol ε[2,18]. The switch of pols occurring during leading and lagging strand synthesis is primarily controlled by the auxiliary protein Replication Factor C (RF-C) and involves a complex network of interactions among pol-prim, RF-C, pol δ and RP-A[25,26,27].

The number of RNA-DNA hybrid molecules required for initiation of leading strand synthesis corresponds to the number of replicons in the genome that in human cell may be approximately $4–8 \times 10^4$. Since pol-prim must synthesize a primer for each Okazaki fragment approximately 200 nt long, the number of initiation events during discontinuous synthesis of the lagging strand in mammals is about $2 \times 10^7$. These numbers raise the still unsolved question on how pol-prim selects the sites for the initiation events. Interaction of pol-prim with other proteins of the replication machinery almost certainly influences the site of leading strand initiation at chromosomal origins[18]. Conversely, the mechanism controlling the selection of the sites for

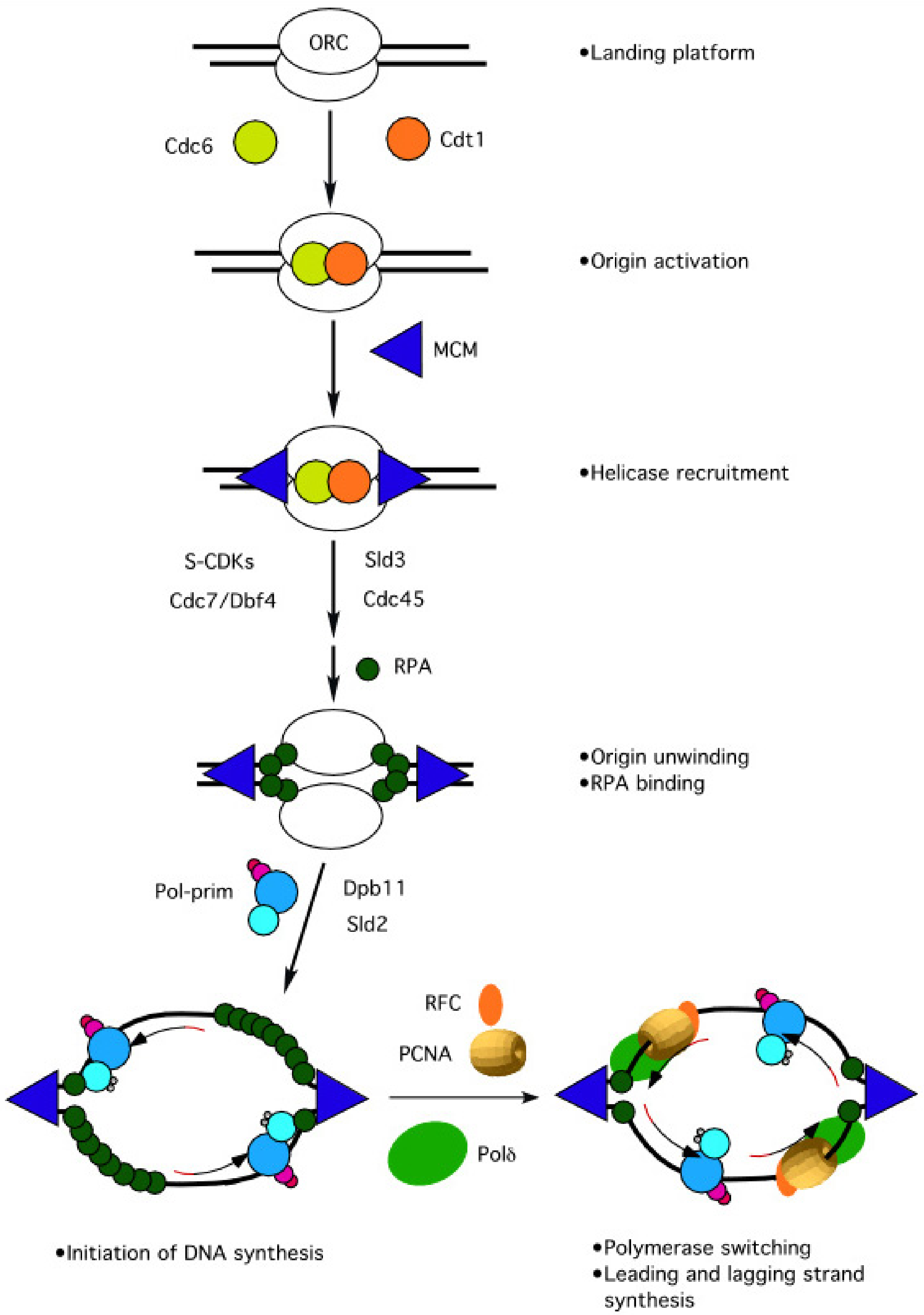

**FIGURE 2**. The assembly of the replication machinery at a yeast origin. The names of the indicated factors corresponds to the components identified in *S. cerevisiae*, even though most of them are common in all eukaryotic cells (see text).

Okazaki fragment initiation is unknown and can be influenced by: (1) specific protein-protein interactions coordinating leading and lagging strand synthesis, (2) topological constraints between the DNA and the replication apparatus, (3) the stochastic presence of a stretch of pyrimidines which appear to be the preferred pol-prim templates.

Pol-prim catalyzes the synthesis of RNA-DNA primers in a minimum of five subsequent steps: template binding, NTP binding, dinucleotide formation, extension to a functional RNA primer, and primer transfer to the pol α catalytic site for elongation. Pol-prim binds to ss-DNA and can slide on it before NTP binding, suggesting that the enzyme does not need to synthesize a primer at the site it first binds the template DNA[28]. On a ss-DNA template pol-prim shows a strong preference for deoxypirimidine polymers with a minimum chain length of 5–10 residues[29]. However, on templates containing a mixture of purines and pyrimidines, pol-prim does not initiate synthesis randomly, but prefers certain sites[30]. After binding the template, the primase subunit must next bind two NTPs to catalyze the formation of a dinucleotide. This appears to be the rate-limiting step: dinucleotides are synthesized at a rate of 0.003 $s^{-1}$ and are then rapidly extended to full-length RNA primers 7–10 nt long[31]. Interestingly, NTPs concentration also influences the sites of initiation: in fact, at low NTPs concentration pol-prim is free to slide along the DNA until it comes to a preferred pyrimidine-rich sequence, while at high NTPs concentration pol-prim initiates synthesis at the first potential site found after DNA binding[28]. An interesting feature of pol-prim is its ability to count the length of the RNA primer elongated by pol α. Counting is not simply the consequence of the presence of a unit-length primer bound to the primase subunit since, in the absence of the dNTPs, pol-prim continues to synthesize RNA primers in modal increments of the original 7–10 nt RNA primer up to 40–50 nt[32,33]. Under normal conditions, after a unit length RNA primer has been generated, subsequent primase activity is inhibited until the primer is either elongated by pol α or dissociates from primase. An intriguing possibility is that synthesis of a unit length primer acts as a termination signal to the primase activity, allowing the 3’OH of the primer to be used by the pol α catalytic site; i.e., primers smaller than 7 nt do not allow the functional switch from RNA to DNA synthesis to produce the final RNA-DNA hybrid synthesized by pol-prim.

## REGULATION OF POL-PRIM DURING THE CELL CYCLE

The expression of human pol-prim genes is upregulated at the level of transcription when cells leave a quiescent state to enter the mitotic cell cycle, but the amount of pol-prim transcript does not fluctuate once mammalian cells are actively cycling[34].

Conversely, in budding yeast, the genes coding for the pol-prim subunits are periodically transcribed at the G1/S boundary, together with several other DNA synthesis genes, due to the presence of the MCB box and the action of the MBF transcription factor[35]. However, the pol-prim subunits are very stable proteins whose level is not rate limiting for DNA synthesis, since yeast cells can undergo several cell divisions, even when the level of these polypeptides drops well below the physiological amount[36]. Therefore, the onset of DNA synthesis during the mitotic cell cycle does not seem to require periodic transcription of the pol-prim genes at the G1/S boundary.

Analogously, the assembly of the pol-prim complex is not restricted to S phase[37] and, therefore, does not seem to be a rate-limiting step in controlling S phase entry. However, physical interaction between p180 and the B subunit may cause a conformational change required for proper B subunit modification[37]. In fact, mammalian and yeast pol-prim complexes have been shown to be phosphorylated in a cell cycle dependent manner[38,39]. In budding yeast, the B subunit is phosphorylated early in S phase, while dephosphorylation occurs when cells exit from mitosis. In human cells both the pol α and the B subunit polypeptides are phosphorylated, and it has been shown that cyclin A-Cdk2 differentially controls SV40 origin-dependent initiation activity of pol-prim *in vitro*[40]. The complex is maximally active when the B subunit is phosphorylated by cyclinA-Cdk2 and the N-terminus of pol α remains unmodified[41]. Metabolic labeling of primate cells revealed the existence of phosphorylated and hypophosphorylated pol-prim populations that are distinguishable by specific monoclonal antibodies[42]. Cell cycle studies showed that the hypophosphorylated form is associated in a complex with serine/threonine phosphatase 2A and cyclin E-Cdk2 in G1, whereas the phosphorylated enzyme is bound to cyclin A-Cdk2 in S and G2. Similarly to what previously suggested for yeast cells[3], it is possible that the hypophosphorylated pol-prim initiates DNA replication at origins, while the phosphorylated form is involved in lagging strand replication. Accordingly, it has been found in yeast that pol-prim undergoes a cell cycle regulated association with chromatin and that only unphosphorylated B subunit is chromatin-bound[43]. However, dephosphorylation of the B subunit is not sufficient to drive pol-prim association to chromatin which, requires, at least in part, one or both of the primase subunits. Interestingly, the recruitment of pol-prim to chromatin during G1 is blocked by yeast Cdk1 but does not require Cdc6, suggesting that, besides pre-RC formation, there is a second component or pathway in DNA replication which is reset in mitosis by inactivation of Cdk1[43].

## POL-PRIM AND THE CELLULAR RESPONSE TO DNA DAMAGE

Checkpoints are a set of genetically controlled mechanisms that actively delay a cell cycle event until a previous one has been properly completed[44]. The concept that these surveillance mechanisms provide cells with some extra time to properly respond to cell cycle perturbations is appropriate for some checkpoints, like the G2 DNA damage response or the S-M checkpoint, that prevent entry into mitosis when DNA is damaged or DNA synthesis is underway. However, this assumption is not completely pertinent for the intra-S DNA checkpoint that is activated by DNA damage or dNTPs depletion during DNA replication. In fact, S phase perturbation does not cause an arrest of the replication process, but DNA synthesis proceeds in the presence of damage although at a reduced rate[45], suggesting that time is probably not the essence of the intra-S checkpoint. We have previously proposed that the replication delay observed in response to DNA damage might be due to variation of the replication process that makes it inherently slower[46]. This modification would make the replication machinery capable of dealing with DNA lesions through a complex interplay between DNA replication, repair and recombination. A corollary of this interpretation is that the replication machinery itself is likely to be one of the targets of the checkpoint pathway. Indeed, the checkpoint response is essentially a signal transduction pathway. Therefore, three main classes of checkpoint factors can be envisioned: (1) sensors that detect DNA damage or replication blocks, possibly generating a signal; (2) transducers that will relay this signal; (3) effectors that act on targets interfering with cell cycle mechanisms or establishing dynamic interconnections among different DNA transactions. In this scenario, DNA replication proteins can possibly act as sensors, targets, or both. One of the first indications of a connection between the replication machinery and checkpoint response came from the discovery that the *Schizosaccharomyces pombe* Cds1 was a suppressor of a pol α temperature-sensitive (ts) mutant[47]. Cds1 is the *S. pombe* homolog of Rad53 (in *S. cerevisiae*) and Chk2 (in human cells), a set of protein kinases acting as central transducers of the checkpoint response in all eukaryotic cells analyzed so far[46]. After that initial observation, several studies connected a number of replication proteins (pol-prim, pol α, RP-A, RF-C, and others) with the checkpoint pathway. Most of these observations were obtained in *S. cerevisiae* and *S. pombe* by analyzing the phenotypes associated to mutations in genes coding for replication factors, or by testing the dependency of the phosphorylation state of critical replication proteins on the activity of checkpoint kinases. For example, it was found that the *pri1-M4* mutation in the gene coding for the p48 primase subunit of the *S. cerevisiae* pol-prim complex, causes an allele-specific and dominant sensitivity to DNA damaging agents and an inability to slow down the rate of S phase progression[48]. The signal transduction pathway triggered by DNA damage and leading to Rad53 activation was unaffected by the pri1-M4 mutation, suggesting that pol-prim acts downstream of Rad53. This conclusion was also supported by the observation that the cell cycle delay caused by Rad53 overexpression is counteracted by the pri1-M4 mutation. Although primase seems to act downstream of Rad53, pol-prim does not appear to be a direct substrate of Rad53 and, therefore, other factors might mediate the transduction cascade from Rad53 to pol-prim. It has also been found that the phosphorylation state of the pol-prim B subunit is controlled by Rad53 following treatments with genotoxic agents; this mechanism probably involves a transient inhibition of Cdk1 in response to checkpoint activation[49]. We have proposed that checkpoint activation may promote alternative mode of DNA replication by combining the replication machinery with recombination mechanism[46]. In this view, the primary effect of Cdk1 regulation in response to checkpoint activation would be to convert the lagging strand replication apparatus to this alternative replication process. Indeed, a replication by recombination mechanism has been described in *S. cerevisiae* cells, which are able to repair double-strand breaks by using a specialized pathway that requires the action of the pol-prim complex[50].

As briefly summarized above, the S-M checkpoint prevents mitosis when DNA replication is still ongoing or is slowed down by genotoxic agents. Recent results obtained with *Xenopus* egg extracts suggest that activation of the S-M checkpoint directly depends on the physical association of pol-prim with unwound ss-DNA during the replication process[51]. Checkpoint induction does not require new DNA synthesis on the unwound template, but requires RNA molecules synthesized by primase. Therefore, RNA primers seem to be an important component of the signal that activates the S-M checkpoint. This conclusion is consistent with our knowledge of the replication process; in fact, the RNA primers synthesized by pol-prim are transient structures that are normally removed during maturation of the leading and lagging strands of newly synthesized DNA. Therefore, abnormally persistent RNA primers seem to be logical candidates to act as triggers of the checkpoint that prevents entry into mitosis until S phase has been completed. However, the characterization of pol-prim mutants in *S. pombe* suggests that the role of pol-prim in the S-M and intra-S checkpoints may be even more complicated[52,53]. In *S. pombe* there are two effector kinases called Cds1 and Chk1 that perform distinct, although partially overlapping, roles in checkpoint response[54]. During DNA replication Cds1 kinase is required for cell survival when replication is inhibited by dNTPs depletion or by DNA damage (intra-S checkpoint), while it is dispensable for the S-M checkpoint controlled by Chk1. Chk1 becomes activated by damage during late S phase or G2. A systematic analysis of mutations in the *S. pombe* pol-prim genes indicates that a stable pol-prim complex is required for the activation of the Cds1-mediated intra-S checkpoint, while an optimal-size RNA primer that can be efficiently utilized by pol α seems to be required for the activation of the Chk1 response[53]. Recently, a connection between the stability of the replisome and checkpoint response has been established by 2D-gel analysis of replication intermediates in wild-type and checkpoint defective *S. cerevisiae* cells following dNTPs depletion caused by hydroxyurea (HU) treatment[55]. It was shown that in wild-type cells treated with HU, replication forks slow down but can resume replication once HU is removed. In contrast, in HU-treated *rad53* checkpoint defective cells, replication forks collapse giving rise to abnormal structures that prevent recovery of DNA replication after HU removal. The role of the checkpoint in maintaining the stability of the replication forks under certain genotoxic conditions might also explain the unrestricted firing of late replication origins when DNA synthesis is blocked in checkpoint mutants[55].

## MULTIPLE POL-PRIM INTERACTIONS SUGGEST ADDITIONAL ROLE FOR THE COMPLEX IN DNA METABOLISM AND IN THE CONTROL OF GENE EXPRESSION

With a combination of genetic and biochemical approaches, it has been found that pol-prim interacts with a large number of other proteins. While the physiological significance of some of these interactions[56] is still far to be proven, multiple interactions are expected for a protein complex playing a central role in several DNA transactions. For instance, the peculiarity of pol-prim being the only cellular pol capable to initiate DNA chains *de novo*, suggests an involvement of this complex not only in the initiation of replication at chromosomal origins, but also in the maintenance of particular chromosomal regions, such as the telomeres. Given the limitations of conventional pols that are able to synthesize DNA only in the 5' to 3' direction, it was predicted that removal of the terminal RNA primer will cause the loss of genetic information at each round of replication, causing chromosomes to shorten every cell cycle. This paradox was potentially resolved by the discovery that telomerase can catalyze the addition of telomeric repeats at the 3' end of a telomeric sequence and pol-prim can start the synthesis of the opposite strand to generate new complete telomeres[57]. The role of pol-prim in telomere DNA replication has been recently proved by the development of an assay capable of monitoring the addition of telomeric sequences onto a *de novo* telomere created i*n vivo*[58], supporting the old-standing notion that mutations in the pol α gene affect telomere length[59]. Moreover, a two-hybrid screen, confirmed by coimmunoprecipitation studies, demonstrated that pol α interact with Cdc13, a telomere-binding protein playing critical roles in telomere metabolism[60].

Extensive studies in *S. cerevisiae* have suggested that DNA replication is important for repression of the silent mating type loci HML and HMR, even though recent work indicates that silencing can be uncoupled from replication[61]. In the fission yeast *S. pombe*, transcriptional silencing at the mating-type region, as well as silencing at centromeres and telomeres, is epigenetically controlled and results from the assembly of higher order chromatin structures. Chromatin proteins associated with these silent loci are believed to promote inheritance of the silenced state during cell division. In particular, the chromodomain protein Swi6 seems to be the most important determinant of the epigenetic imprint. Recently, it has been shown that mutations in the pol α gene alleviate or abrogate silencing at the mating type or centromere and telomere loci in *S. pombe*. Moreover, Swi6 and pol α physically interact both *in vivo* and *in vitro* [62,63]. These findings indicate that pol-prim may perform an imprinting function in establishing a chromatin state that is competent to recruit heterocromatin components, such as Swi6. Because both pol α and Swi6 are important and conserved components of DNA replication and heterocromatin assembly, then pol α-Swi6 interaction might be conserved during evolution and may serve as a model for gene regulation during development in higher eukaryotes. Interestingly, *S. pombe* cells seem to utilize the pol-prim complex and the intrinsic asymmetry of the DNA replication machinery, i.e., the difference between the replication mechanisms of the leading and lagging strands, to establish an asymmetrical mating-type switching pattern[64]. In this context, it has been suggested that the control of the direction of replication at specific chromosomal loci may represent a general mechanism for the establishment of differentiation and development also in multicellular eukaryotes[65].

## CONCLUSIONS

Pol α was the first discovered DNA polymerase in eukaryotic cells and for several years it was considered as the only pol involved in DNA replication. Later, other replicative pols were

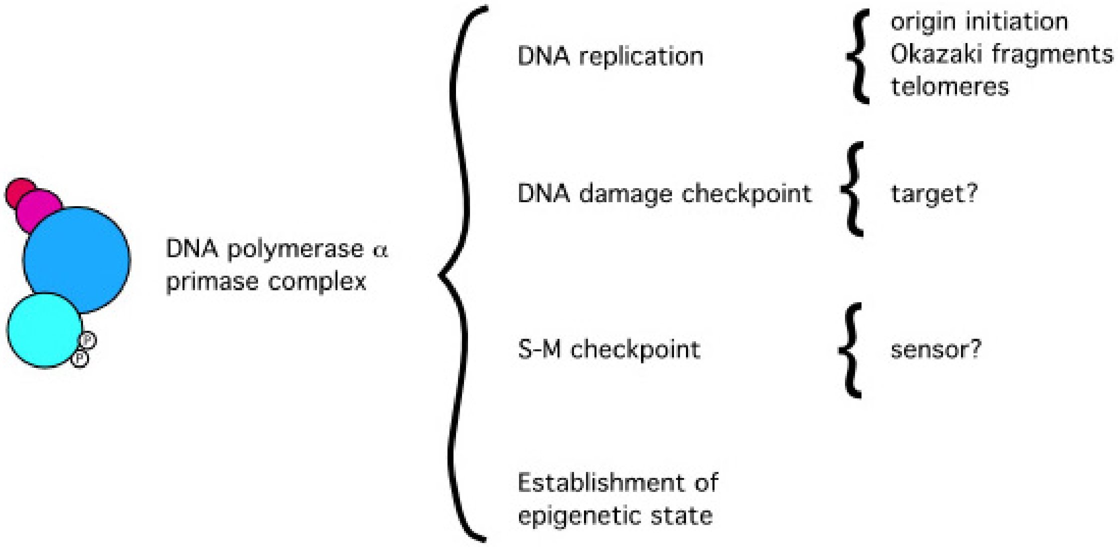

**FIGURE 3**. Functions of the pol-prim complex in DNA metabolism.

identified, but pol α remained unique because of its association with DNA primase, and the existence of the pol-prim complex explained several aspects of the chromosomal process (Fig. 3).

The recent findings that pol-prim is involved in other relevant cellular processes, such as the response to DNA damage and the epigenetic control of chromatin structure, indicate that the variety of functions played by pol-prim in many DNA transactions are far from being completely understood.

## ACKNOWLEDGMENTS

Work in our laboratory was supported by grants from Associazione Italiana per la Ricerca sul Cancro, Cofinanziamento MURST-Universita' di Milano, MURST (5%) Biomolecole per la Salute Umana, CNR Target Project on Biotechnology , CNR Agenzia 2001.
